# Parenclitic networks for predicting ovarian cancer

**DOI:** 10.18632/oncotarget.25216

**Published:** 2018-04-27

**Authors:** Harry J. Whitwell, Oleg Blyuss, Usha Menon, John F. Timms, Alexey Zaikin

**Affiliations:** ^1^ Chemical Engineering, Imperial College London, London, United Kingdom; ^2^ Wolfson Institute of Preventive Medicine, Queen Mary University of London, London, United Kingdom; ^3^ Institute for Women's Health, University College London, London, United Kingdom; ^4^ Department of Mathematics, University College London, London, United Kingdom

**Keywords:** parenclitic, network, ovarian cancer, biomarker, serum

## Abstract

Prediction and diagnosis of complex disease may not always be possible with a small number of biomarkers. Modern ‘omics’ technologies make it possible to cheaply and quantitatively assay hundreds of molecules generating large amounts of data from individual samples. In this study, we describe a parenclitic network-based approach to disease classification using a synthetic data set modelled on data from the United Kingdom Collaborative Trial of Ovarian Cancer Screening (UKCTOCS) and serological assay data from a nested set of samples from the same study. This approach allows us to integrate quantitative proteomic and categorical metadata into a single network, and then use network topologies to construct logistic regression models for disease classification. In this study of ovarian cancer, comprising of 30 controls and cases with samples taken <14 months to diagnosis (*n* = 30) and/or >34 months to diagnosis (*n* = 29), we were able to classify cases with a sensitivity of 80.3% within 14 months of diagnosis and 18.9% in samples exceeding 34 months to diagnosis at a specificity of 98%. Furthermore, we use the networks to make observations about proteins within the cohort and identify GZMH and FGFBP1 as changing in cases (in relation to controls) at time points most distal to diagnosis. We conclude that network-based approaches may offer a solution to the problem of complex disease classification that can be used in personalised medicine and to describe the underlying biology of cancer progression at a system level.

## INTRODUCTION

Personalised medicine is hailed as the next significant step in the treatment and monitoring of diseases. Some cancer treatments, for example Trastuzumab in HER-2 positive breast cancer, are already tailored to individuals based on personal expression data [1]. Given the rate at which technology is advancing for high-throughput molecular analysis, it is inevitable that samples will be routinely taken from a patient and analysed by a range of ‘omic technologies [2]. Whilst this will allow truly personalised medicine, there will be huge challenges in analysing large amounts of multi-dimensional and longitudinal data. Given the technological advances of high-throughput, multi-omic technologies, a conceivable ideal would be to take all available data and identify changes that indicate an early stage malignancy or accurately predict the formation of such. The cancer genome atlas has shown that there are many possible combinations of changes responsible for the onset of particular cancer types [3]. Hence there is a need for a procedure that considers the changes in a system as a whole, i.e. a network biomarker. Herein, we present an algorithm for generating parenclitic networks that are optimised towards biomarker identification. The technique we present can also inform on the underlying biology [4], as well as provide network models for prediction. Thus, we believe this could be an important progression in the advancement of personalised medicine.

Parenclitic networks, first described by Zanin *et al*. in 2014 [5, 6], are established by determining differences between pairs of analyte measurements (protein, mRNA etc.) in a control data set and individual case samples. If the difference is above a threshold, then a linkage between the analytes is created. This is repeated for every pair of analytes to generate a network. In [5], the differences were calculated by plotting a linear regression through a control population for a specific analyte pair and then calculating the perpendicular distance from the line of regression to the sample point for the same pair. We first show that a better approach, at least from the perspective of constructing a network-biomarker, is to use 2-dimensional kernel density estimation (2DKDE) as an underlying model for the control distribution. The difference is determined by the area under the density distribution in the controls for the sample marker-pair, thus if the sample marker pair lies in a region of low density, the area under the density distribution will be larger, and the inferred distance is greater. We have also developed the network approach to allow the inclusion of categorical as well as continuous data, allowing testing of cancer risk variables on the networks. In our approach, we take all available information and reduce it to network topological features that are then built into logistic regression models.

We apply the approach to the prediction of ovarian cancer (OC) using multiple protein measurements made in pre-diagnosis serum samples from a cohort of type II OC cases and matched controls. Ovarian cancer is the 6th most frequently occurring cancer in women and 10 year survival is only 35% for both Type I and Type II cancers combined [7]. Type II cancers are much more aggressive than Type I, and are responsible for the majority of deaths. Cancer antigen 125 (CA125) is the current best biomarker for OC, however it is only predictive in late stages of the disease when survival is between 5–19% [8]. A recent report from the UK Collaborative Trial of Ovarian Cancer Screening (UKCTOCS), which used serial serum CA125 measurements, showed that whilst there was no significant benefit to mortality, there was a stage shift at diagnosis [9]. This supports that screening for OC may be a sensible approach in the future, but better models are required for earlier detection that will translate into improved mortality.

Herein, we employ parenclitic networks for disease prediction in a small cohort of ovarian cancer cases and controls at two time points with respect to clinical diagnosis. We also generate average (mode) networks for each time group that highlight differences in the protein-networks between cases and controls at these two time points.

## RESULTS

**Figure 1:**
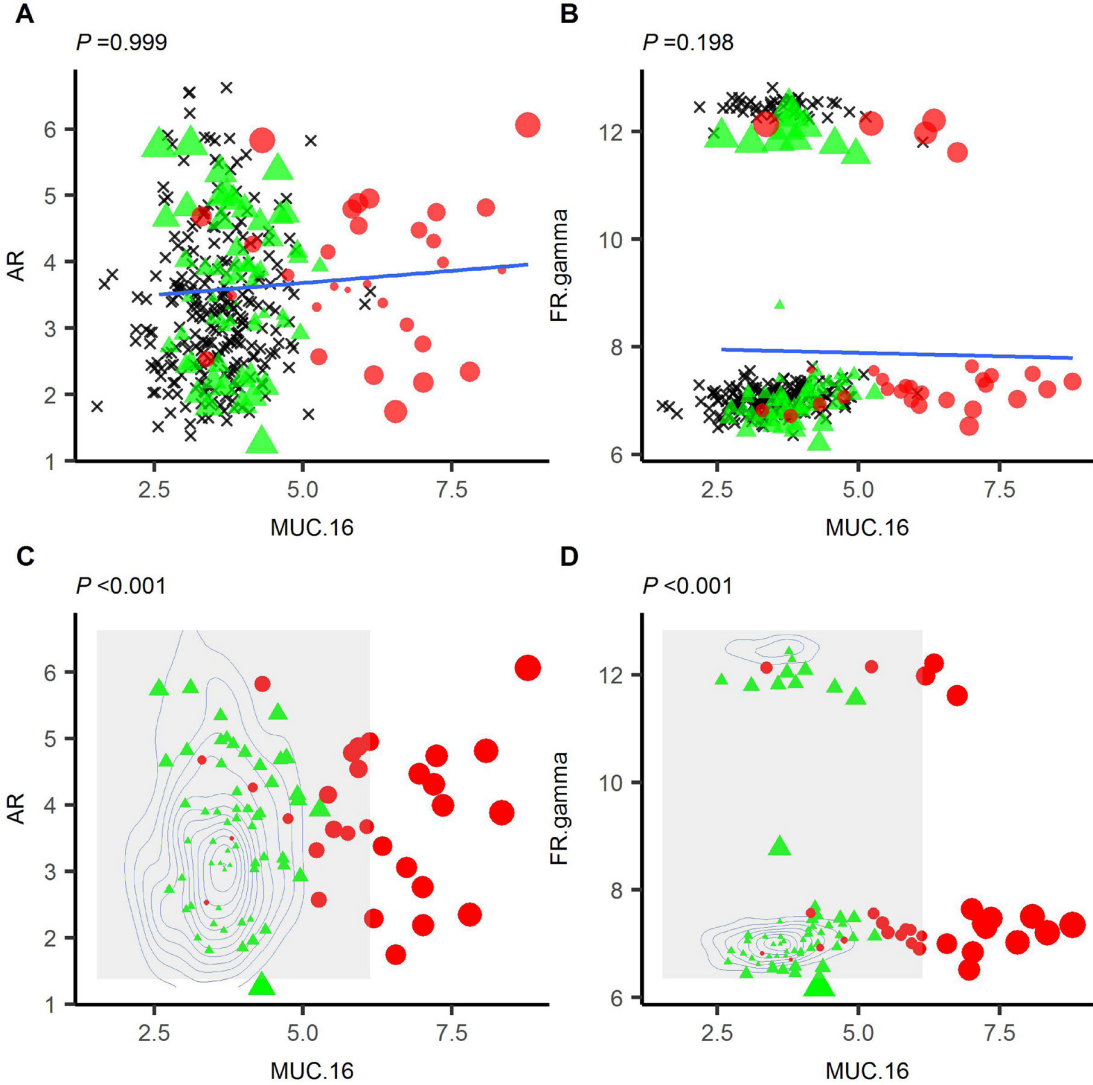
2D-Kernal density estimation-based distance can differentiate cases (red circle) and controls (green triangle) where linear-regression based distance cannot Linear regression was plotted using a control set (black crosses) for MUC16 vs AR (**A**) or MUC16 vs folate receptor gamma (**B**). Cases and controls are then overlaid and the perpendicular distance from each point determined. It was not possible to differentiate cases from controls using the linear regression (*P* = 0.999 or 0.198 for AR or folate receptor gamma respectively). 2DKDE estimation of the same distributions was performed (**C** and **D**) and distance calculated for each case and control based on the density of the underlying distribution. In both cases, it was possible to differentiate cases and controls in this manner.

Linkages between analytes in an individual are based on how the analyte pair is predicted to deviate from a control population. Zanin *et al.* [5] demonstrated that this can be performed by plotting a linear regression through a sample set of data and, for each individual, calculating the perpendicular distance (normalised by standard deviation (z-score)) from the regression for each analyte pair. To implement this in a general algorithm, one must assume that all pairs of analytes will be both correlative and follow a linear model. However, in biological samples, this is often not the case. For example, in Figure 1, we have plotted MUC16/CA125, an OC biomarker, against androgen receptor or folate receptor gamma, which form a non-correlative or a bimodal distribution respectively (Figure 1A, 1B). In neither case was the average distance able to differentiate between cases and controls. When repeating for all combinations of markers with MUC16, the mean *P*-value was 0.42 (SD = 0.31). We have overcome this by using 2-dimensional kernel density estimation to predict the density of two markers in a non-cancer control population. Sample deviation is determined based on the estimated density for the analyte pair (see methods and Supplementary Information (SI)). For the same combinations of markers, we were able to differentiate between cases and controls (Figure 1C and 1D) with a mean *P*-value of 3.17 × 10^-7^ (SD = 2.01 × 10^-6^). Therefore, we employed 2DKDE in our algorithm for linkage assignment.

**Figure 2:**
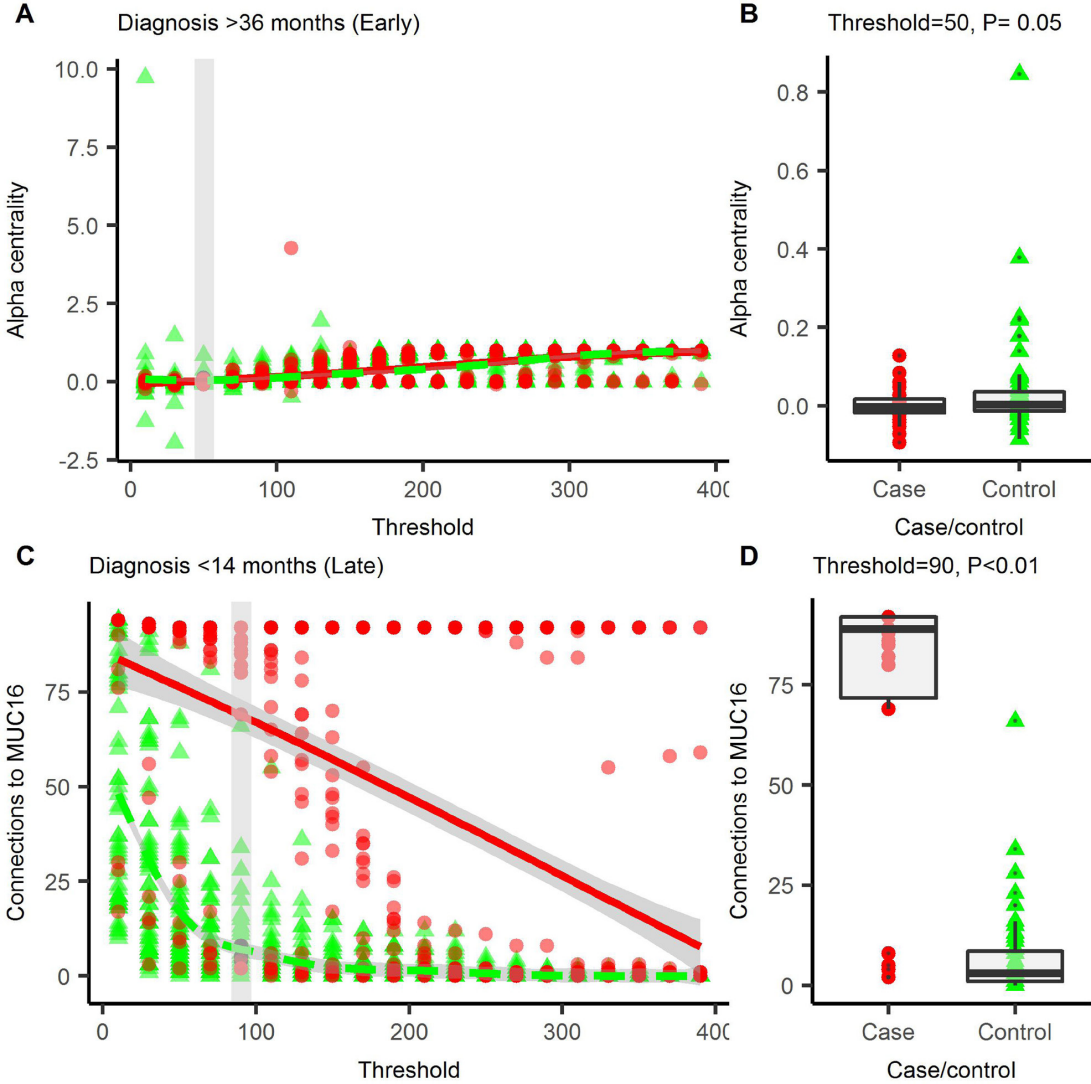
Parenclitic networks are generated across a range of thresholds At each threshold, the network is described using a number of topological indices, the index value at each threshold is presented for connections to MUC16 (**A**) and alpha-centrality (**C**), which featured in prediction models for OC in the early, and late groups respectively. For each index, the smallest threshold that gives the greatest degree of differentiation between cases and controls was used to build the logistic regression models. For connections to MUC16, this was at a threshold of 90 (**B**) and for alpha-centrality, 50 was optimal (**D**). Red circles = cases, green triangles = controls, solid line = trend line. In A and C, the grey box is the optimal threshold; this data is shown in B and D.

Preliminary investigations with a longitudinal, synthetic data set modelled on CA125 (see Supplementary Information 2), showed that topological features of the networks can be used to detect changes within the data set (see Supplementary Figures 1, 2) at a given threshold. Not all topological features are maximally discriminative between cases and controls at the same threshold so we cycle through a number of thresholds to iteratively determine the optimum for each topological feature (Figure 2A and 2C, Supplementary Figures 3, 4). These descriptors can be combined into a multi-parameter logistic regression for disease prediction. We tested this procedure in an OC data set comprising type II OC cases and controls (*n* = 30), where each individual has two serum samples taken <14.5 months (late, *n* = 30) or >34.5 months (early, *n* = 29) prior to diagnosis. Protein quantification for each sample was performed by proximity extension assay for a panel of 92 cancer-related proteins (Olink Oncology II panel). A second data set, comprising 120 controls was also assayed with the same panel and used to generate the kernel density estimates (for further description of both data sets, see methods and Supplementary Tables 1-2). The best model for each time group generated using the parenclitic methodology was then compared with logistic models generated using the raw data (raw data logistic regression, RDLG) after Monte Carlo cross-validation. At a specificity of >98%, the best parenclitic model had a higher sensitivity in both early and late groups (Table 1).

**Table 1:** Area under the ROC curve (AUC), specificity and sensitivity of the best performing parenclitic and RDLG models for early (>34.5 months to diagnosis) and late (<14.5 months to diagnosis) groups

Group	Type	Model	AUC	Specificity	Sensitivity
Early	Parenclitic	-7.44 (Alpha-centrality)	0.624	1	0.189 ± 0.124
		-0.16 (Pill use)			
	RDLG	-2.09 (GPC1)	0.663	1	0.163 ± 0.087
Late	Parenclitic	0.07 (Connections to MUC16)	0.904	1	0.803 ± 0.077
	RDLG	2.14 (MUC16)	0.904	1	0.767 ± 0.082

Models were generated using all combinations of 1, 2 or 3 markers and following cross-validation the best models were selected based initially on sensitivity and secondly on AUC. Specificity was set to be >98% and the maximum is reported for the given sensitivity. Models were generated by logistic regression of either the parenclitic topologies or raw data and model coefficients are provided.

**Figure 3:**
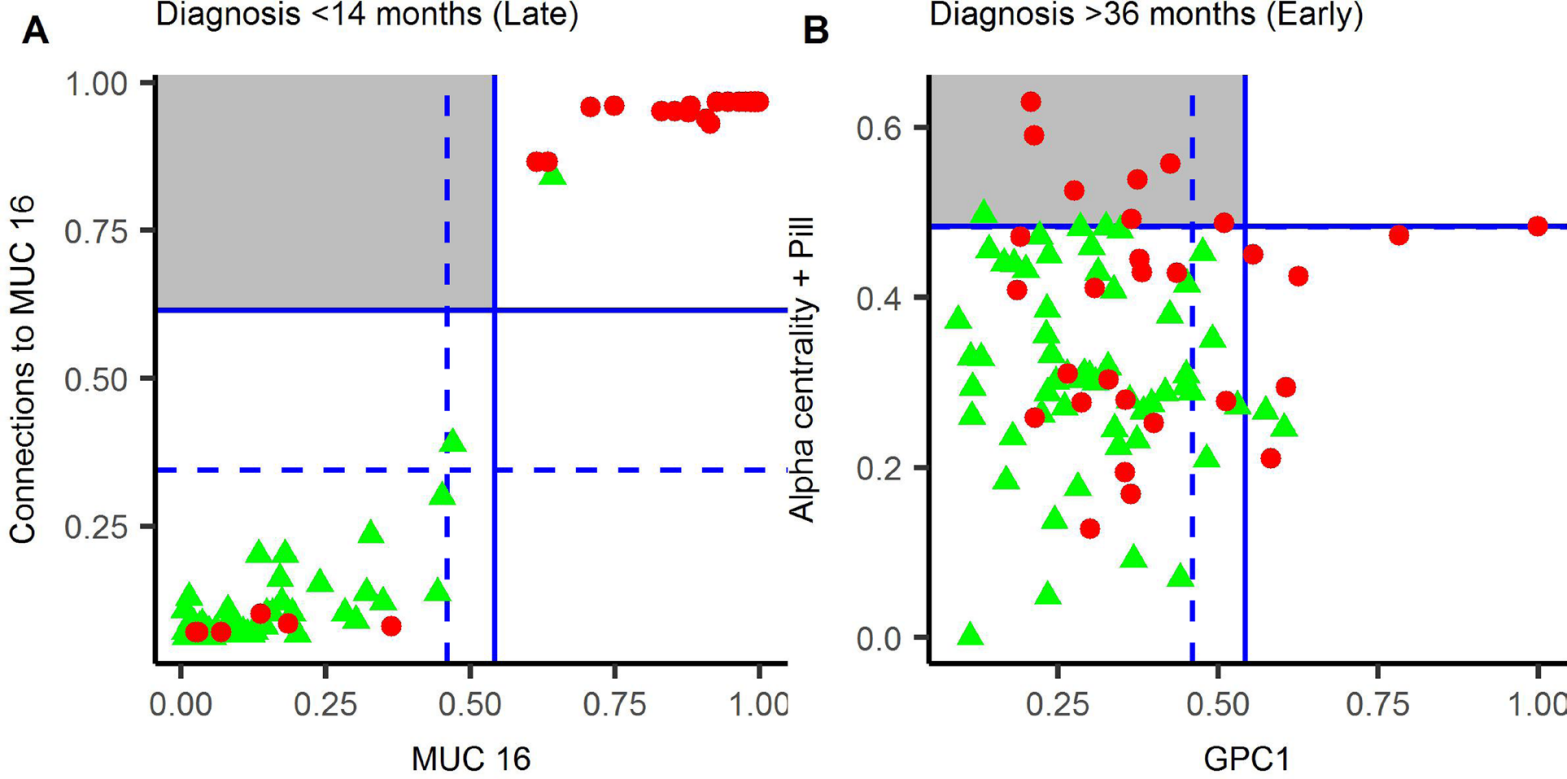
Parenclitic-based models versus RDLG-models for late (**A**) and early (**B**) pre-diagnosis groups. The predicted value for each model is plotted with horizontal and vertical lines representing a diagnostic threshold giving 95% (dashed line) or 98% (solid line) specificity. Cases are represented by red circles and controls by green triangles, hence cases in the top left quadrant (grey square) were detected by the parenclitic-model only and cases in the bottom right quadrant by RDLG-models only. Points in the top right quadrant are detected by both models, OCP = oral contraceptive pill.

In the late group (<14 months to diagnosis), the best parenclitic model used “number of connections to MUC16” (Figure 2B), with the RDLG model using the raw values for MUC16. Whilst the sensitivity of the parenclitic model (80.3%) was higher than the RDLG model (76.7%), suggesting that the parenclitic approach favours discrimination, there was no improvement on case/control discrimination within this data-set. (Figure 3A).

In the early group (>34.5 months to diagnosis), the parenclitic model comprised of alpha-centrality (Figure 2D) and oral contraceptive pill (OCP) use (as a categorical variable), whereas in the RDLG model, glypican 1 (GPC1) was used. In this group, the higher sensitivity achieved for the parenclitic model (18.9%) compared to the RDLG model (16.3%) translated into 8 predicted cases in the parenclitic model, compared to 6 in the RDLG model (Figure 3B). Furthermore, of these, only 1 was diagnosed by both tests, suggesting that the parenclitic and RDLG models are identifying orthogonal features and combining them in a decision tree may provide improved discrimination at early time points.

**Figure 4:**
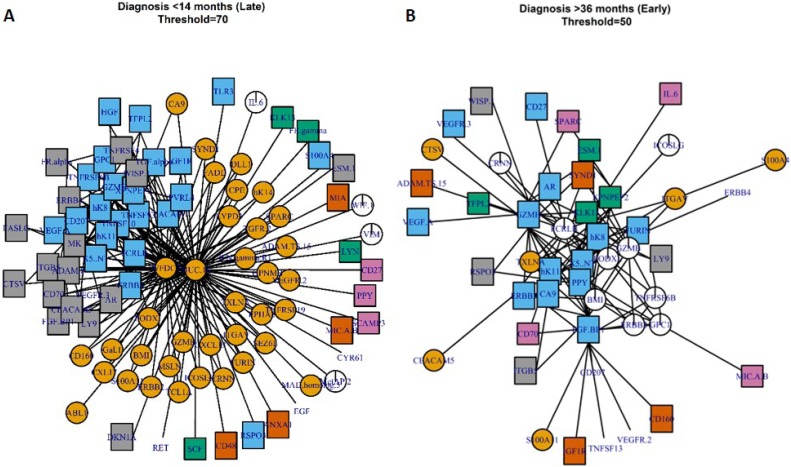
Modal-networks represent the average networks for individuals at <14.5 months to diagnosis (**A**) or >34.5 months to diagnosis (**B**). Colours and shapes represent community membership as determined from clustering by edge-betweenness topology.

Parenclitic networks can also be used to inform on changes within biological systems without the arbitrary cut off of a *P*-value or fold change. Thus, we generated a modal network to investigate differences between cases and controls and between the early and late OC groups. The modal network was produced by generating networks for each individual and taking the modal state of a linkage – i.e. if the modal state between any two markers was connected, they were connected in the modal-network. Networks were generated across a range of thresholds with community membership identified based on edge-betweenness clustering. Well connected markers are found in the centre of clusters and these “hub-centres” represent proteins that are the most different between cases and controls (Figure 4). In samples from the late group, i.e. closest to diagnosis, WDCF2 (HE4) and MUC16 (CA125) were clearly at the centre of the largest hub. Midkine (MK) was also well-connected in a subsidiary cluster, although it is not a clear hub-centre (Figure 4A). In samples taken furthest from diagnosis (early, >34.5 months), granzyme-H (GZMH) and fibroblast growth factor binding protein 1 (FBF BP1) are well-connected centres and thus may indicate involvement in the onset of OC or response to tumorigenesis (Figure 4B).

Whilst GO-term enrichment analysis shows that there is some enrichment for serine proteases within the group (data not shown), the protein pool is small and biased towards “cancer-related” proteins and consequently we do not hold much weight by this observation and have not investigated this further.

## DISCUSSION

Parenclitic networks have been utilised for a number of approaches including both biological discovery [5] and cancer detection [4, 6]. In these cases, the methodology underlying edge/connection creation within the network has relied on multiple linear regression models. In biological data sets, the assumptions for performing a linear regression are not always met. In Figure 1, we provide examples of non-correlating and bimodal distributions in which distance from a linear regression between a disease-predictor (MUC16) and a non-predictor (AR, folate receptor gamma) is not able to differentiate between cases and controls. The method we have developed describes the data with a greater degree of fidelity by using 2DKDE and thus can achieve a better estimation of distance between an individual and a population. Indeed, we distinguished between cases and controls for all pairs of distributions that included MUC16. The 2D density matrices require a large number of samples to describe the distribution accurately [10, 11], hence in this study, we used 249 independent control samples for deriving the density matrices and we would envisage that with fewer samples, this methodology would not work as effectively. This is being further investigated with synthetic data.

MUC16 (CA125) is currently the best diagnostic marker for OC, however, the specificity and sensitivity of the marker is hampered by elevated expression in a number of benign diseases and other cancer types [12]. Whilst the marker may be elevated at early stages of the disease, recent large studies of annual screening showed there was no improvement in mortality for those diagnosed from elevated CA125 or otherwise [13, 14]. In using a network approach, we hoped to be able to pick up on subtle changes in protein expression prior to diagnostic levels of CA125 [3]. As the data set was small, we aimed to avoid overfitting by limiting the number of possible markers in a logistic regression to 3 and cross-validated all results by the Monte Carlo method. At both time points, the parenclitic-based model had higher sensitivity than the best model generated from standard linear regression (Table 1). In the late group (close to diagnosis), this increase was marginal and the network-topological descriptor that features in the model was dependent on the expression of MUC16. Therefore it is unlikely that at time points closest to diagnosis we would be able to improve greatly upon current models that use this marker. In the networks, MUC16 was very highly connected close to diagnosis in cases, and this was also evident in the modal network (Figure 4A).

Mortality from OC is improved by diagnosis at earlier stages, therefore a test that can detect OC earlier is potentially of significant impact. In the raw data, we found that glypican-1 (GPC1) was the most predictive marker in the early (>34.5 months to diagnosis) group (sensitivity = 16.3%). GPC1 was recently identified as elevated in exosomes prepared from the serum of patients with pancreatic cancer [15] and may be involved in disease progression as a mediator of angiogenesis [16], although interestingly, a reduction of GPC1 was found to be predictive of disease in this case. Whilst the AUC was lower for the parenclitic model than the RDLG model, we did show improved sensitivity (sensitivity = 18.9%), a more clinically relevant measure, within this group and were able to detect a greater number of cases than the RDLG model. The best model found through the network topologies was reduced alpha-centrality (Figure 2D) combined with use of contraceptive pill as a categorical variable. Alpha-centrality is a universal measure of eigenvector centrality and is related to number of degrees (connections). Use of the contraceptive pill is known to reduce the risk of OC, particularly in non-BRCA1/2 mutation carriers [17, 18].

In the early group (>34.5 months to diagnosis), we showed that the best parenclitic model and best RDLG model detected different cases, with only a single patient overlapping between them (Figure 3B). This raises the possibility of employing a decision-tree based test, and indeed, doing so within this data set would give a sensitivity of 48.3%. Whilst we do not have sufficient statistical power within this data set to validate this finding, it warrants further investigation.

Early stage OC is often asymptomatic, or symptoms are easily confused with other ailments and therefore it is usually diagnosed at a later stage when symptoms are more pronounced. In order to diagnose OC at earlier stages, a screening program coupled with surgery and chemotherapy may be successful [19]. With screening comes the opportunity to increase the power of statistical models for disease prediction by exploiting longitudinal models that consider an individual's baseline. Combining parenclitic methodologies with longitudinal data analysis may provide further sensitivity. In our synthetic data-set, modelled on CA125 in UKCTOCS, we observed immediate changes in a number of topological indices at the initiation of “cancer” (SF 2). In future, we hope to investigate longitudinal changes in the networks using real data-sets from UKCTOCS.

The power of parenclitic analysis is that as well as being compatible for use in predictive models, it may also be possible to investigate the underlying biology of cancer progression [5]. The advantages with using our parenclitic-distance measure over conventional techniques is that it avoids the use of arbitrary fold-change or *P*-value cut-offs [20]. On the other hand, we do have to make a cut-off for distance thresholds and, although this is somewhat subjective, it is done with the advantage of seeing all the data represented in a single figure, allowing the researcher a greater level of insight when selecting the degree of network complexity. Model network analysis of OC in late stages highlighted MUC16 (CA125) and WFDC2 (HE4) as being responsible for most of the differences within the network (Figure 4A) and this is in line with these being the best reported serological markers of OC [21–24]. In the late-group network, there are two other clusters (blue or grey squares) in which MK is well-connected, although not an obvious hub-centre. Midkine is a growth factor with a diverse role in cell growth and development [25] as well as being a marker and potential mediator of cisplatin resistance [26, 27]. That all three proteins are well-connected within 14 months to diagnosis, but not at 36 months, suggests their involvement in the progression of OC in response to early oncogenic events. Inhibition of MK in a number of tumor cell lines leads to reduced growth [28] and there is growing interest in this protein in a number of applications. The function of MK in ovarian cancer specifically is not well-understood, although its epigenetic modification in ovarian cancer cell lines has been linked to acquired cisplatin resistance [29]. Perhaps further investigation into the function of MK in ovarian cancer would be prudent.

In the early-group modal network, there was no single dominating hub. Whilst there was some enrichment of serine proteases in the connected proteins, the protein pool is small (93 proteins) and manually selected as “cancer-related”, therefore, GO-term enrichment analysis provided no useful information. However, GZMH and FGFBP1 are both well connected within the network, indicating their differential expression between cases and controls. GZMH has been reported as upregulated in some OC-tumor infiltrating leukocytes [30] as well as serologically in breast cancer [31] and this may be indicative of enhanced tumor-specific leukocyte activity in the early stages of disease. FBFBP-1, to the knowledge of the authors, has not been reported as elevated during ovarian cancer, indeed staining could not be seen in histological samples of ovarian tissue held by the human protein atlas (https://www.proteinatlas.org/ENSG00000137440-FGFBP1/tissue/ovary) [32]. It is, however, involved in cell proliferation and angiogenesis and is associated with a number of other cancers [33, 34]. Both GZMH and FGFBP1 would be interesting to investigate further in the context of early OC development.

## CONCLUSIONS

We have developed a novel method to generate parenclitic networks that uses high-fidelity 2D density distributions to measure the differences between co-variables in a data set. Using topological indices achieved better discrimination of pre-diagnosis OC cases and controls than raw data logistic-regression models. Modal-networks showed differences GZMH and FGFBP1 between cases and controls at >34.5 months prior to diagnosis and they may be relevant in the development of OC. Parenclitic networks can be used in biomarkers models and improve upon the sensitivity of simple, linear regression-based models. In the context of ovarian cancer, we were able to detect, with a sensitivity of 18.3% and a specificity >98%, OC in individuals >34.5 months to diagnosis. Interestingly, the parenclitic-based and RDLG models diagnosed non-overlapping cases at early time points and this raises the option of validating a decision-tree based method in a larger cohort. We aim to validate our current models in larger data sets for early detection of OC and postulate that this approach could be exploited in future data-driven personalised medicine.

## MATERIALS AND METHODS

### Sample set

A nested set of OC cases and controls were taken from the multi-modal screening arm of UKCTOCS as part of the Predicting Risk of Ovarian Malignancies, Improved Screening and Early detection (PROMISE) study. UKCTOCS was approved by the Joint UCL/UCLH Research Ethics Committee A (Ref. 05/Q0505/57). Written informed consent was obtained from donors and no data allowing identification of patients was provided. Trial participants at enrolment were post-menopausal women aged 50-74 who had no family history of ovarian cancer. Women subsequently diagnosed with ovarian cancer were identified by cross-referencing with the Health and Social Care Information Centre cancer registry and death codes, with diagnosis confirmed by review of histopathology reports. Paired serum samples from 29 early (>34.5 months to clinical diagnosis) and 30 late (<14.5 months to clinical diagnosis) type II OC cases (mostly high-grade serous) randomised to the multi-modal arm of UKCTOCS. Samples from 30 controls with no history of cancer were matched to cases by age (±5 years), collection date and collection centre. Epidemiological data (OCP use (ever), hormone replacement therapy use (at randomisation), body mass index and age) was available for these women. For more information, see Supplementary Tables 1–2. Single samples from an independent set of 249 non-cancer control women from UKCTOCS were also selected as base controls to build density distributions.

### Serum protein measurements

Ninety two cancer-related analytes were measured in individual serum samples using Olink's multiplex immunoassay Oncology II panel. Based on the proximity extension assay [35], this validated platform uses matched antibody pairs linked to DNA reporters. When binding to their target, the pair gives rise to amplicons which are quantified by RT-PCR, providing high sensitivity and accuracy. Known cancer antigens including MUC16, growth factors, receptors, angiogenic factors and adhesion regulators are measured [36]. Data was returned as log2-transformed normalised expression values.

### Parenclitic network and model testing

For a detailed description of methods, see supplementary information. In brief, each subject/sample is represented by a graph (network) that includes both continuous and categorical data. Edges are set based on how pairs of covariates differ from a cloud of controls (base controls) by determining the distance based on two dimensional kernel density estimation (2DKDE). The greater the deviation of a pair of covariates from the region of greatest density in the base controls, the greater the distance. The inclusion of categorical-continuous data pairs is achieved by calculating the deviation from a set of base controls of the same categorical value. Once all distances are calculated, a threshold is applied so that only edges between deviating covariates remain, such that each subject/sample is represented by a graph of unique topology. Classification is then performed by combining indices of topology into logistic regression models with performance being assessed by sensitivity at >98% specificity and the area under the ROC curve (AUC). The top 5 parenclitic and RDLG models (selected based on sensitivity and then AUC) were further validated by Monte Carlo cross-validation by splitting the data 50/50 100 times. All analysis was performed in R Studio (1.0.143) running R version 3.4.0. Networks were generated using the igraph package (version 1.0.1).

### Modal-network construction

Modal networks were derived by generating parenclitic networks for each sample at a range of thresholds. For each threshold, the networks were combined into a single network where nodes are connected, if they were connected in more than half of the individual networks (i.e. the modal stage of connectedness). The analytes were then clustered using the cluster_edge_betweenness function in the igraph package (version 1.0.1) and community membership determined based on maximum modularity score in the same function.

## SUPPLEMENTARY MATERIALS TABLE AND FIGURES



## Abbreviations

2DKDE: 2-dimensional kernel density estimation (2DKDE)
OC: ovarian cancer
CA125/MUC 16: cancer antigen 125
UKCTOCS: United Kingdom Collaborative Trial of Ovarian Cancer Screening
RDLG: raw data logistic regression
OCP: oral contraceptive pill
ROC: receiver operating characteristic
AUC: area under the ROC curve
