# Supplementary material for: Parenclitic networks for predicting ovarian cancer
Source: Oncotarget. 2018 Apr 27;9(32):22717–26. doi: 10.18632/oncotarget.25216 (PMC5978260; doi:10.18632/oncotarget.25216)
Supplement: Supplementary file 1 [file oncotarget-09-22717-s001.pdf]

Parenclitic networks for predicting ovarian cancer

SUPPLEMENTARY MATERIALS

Supplementary information 1: Algorithm

Building and testing of models in R includes the following steps

1. We subset data including $N_{individuals}$ with N variables/parameters into two sets:

a. set I, a set of controls to construct 2DKDE for all pairs of analytes;

b. set II, a set of controls and cases to build the network and topological models.

2. Training set I is used to construct the 2DKDE matrix filled for every pair of analytes by calling the kde2d function from the Massx package. The size of the matrix is chosen to be $N_{bins} \times N_{bins,}$ $N_{bins} = 16$ but 2DKDE values are known to be independent of this parameter [1]. We generate $N_{bins} \times N_{bins}$ contour matrices.

3. The networks are generated for each individual in a data frame of set II. This network is a graph formed by N nodes/analytes and binary edges between them. Edges can be between continuous-continuous, continuous-categorical, or categorical-categorical parameters (not used in this analysis). In each, graph nodes are formed by analytes, continuous or categorical, and the binary edge is calculated as following:

a. For each individual, the value of the edge between two continuous analytes (i, j), $i, j = 1, ..., N$ forming nodes is calculated from the contourmatrix for this pair of parameters. Here we have two cases:

i. If the sample, i.e., a pair of parameters (x_i, y_j) from data set II corresponding to an individual, is within the matrix of the density estimation, the edge is equal to the volume under the densities that are higher than the density where the sample is within the distribution.

ii. We estimate distance as the normalised ratio between two distances, $\frac{D_{max}}{D_{edge}}$ where $D_{max} = \sqrt{(x_i - x_{max})^2 + (y_j - y_{max})^2}$ and is the distance between the nearest point on the contour matrix $(x_{nearest,} y_{nearest})$ which lies on the straight line between the point with the maximal density $(x_{max,} y_{max})$ and investigated sample point (x_i, j) and is the closest point to (x_i, y_j).

Note that alternatively this distance can be estimated from the linear regression (original version of the parenclitic analysis [2, 3]) or from the Mahalonobic distance [4]. Using the linear regression for each pair of analytes, m_i and m_j, we build a linear regression based on the control group set I:

$$m_j = \alpha_{i,j} + \beta_{i,j} x m_i \quad (4)$$

where $\alpha_{i,j}$ and $\beta_{i,j}$ are regression coefficients. Next we build complete weighted graph for each cancer-positive and negative sample from set II, excluding the control group of set I, such that each vertex corresponds to a particular analyte, and edges are weighted according to

$$w_{i,j} = \frac{|x_j - (\alpha_{i,j} + \beta_{i,j} x_i)|}{\sigma_{i,j}} \quad (5)$$

where x_i and x_j are respective analyte levels for the sample (i, j), and $\sigma_{i,j}$ is the standard deviation of errors in the linear regression model for control objects. In the second case, we implement the Mahalanobis distance [4], which, essentially, measures separation between data sets. In particular, the edge weight is as:

$$w_{i,j} = \sqrt{(x_{i,j} - \mu_{i,j})^T S_{i,j}^{-1} (x_{i,j} - \mu_{i,j})} \quad (6)$$

where, as before, x_i and x_j are analyte levels in an investigated sample, μ_i and μ_j are the analyte levels in the control data set I, $(x_{i,j} = x_i, x_j)$, $\mu_{i,j} = (E(m_i), E(m_j))$ and $S_{i,j} = cov(m_i, m_j)$, where $E(.)$ is the expectation and the $cov(.)$ covariance.

b. Distances between continuous-categorical variables are calculated by generating a density estimation of the continuous data using points from training data set I with the value of the categorical variable equal to the value of the investigated individual. It means that we estimate the deviation only from the corresponding group characterised by the same value of the categorical. Then the distance is calculated as above.

c. Edges between categorical data points are not used in this version but can be potentially calculated from a density estimation of the continuous data by selecting a significant pair of variables (e.g. most predictive pair by AUC) and using the points from training data set I with the value of the categorical equal to these values of the investigated individual. Again in this way we estimate the distance from the group with the same categorical only evaluated on the plane of the most predictive continuous analytes.

d. A threshold is applied to the distances and they are coded 1 (higher than threshold) or 0. A copy of the

network is saved in a new data frame (distance) that contains only those that are greater than the threshold value. This is used for calculating some of the topological indexes later.

4. Each individual is represented by calculating the topological indices for the original and binary graph, both representing the same patient. The number of edges in constructed graphs makes a straightforward machine learning classification task intractable in practice, also manifesting a huge imbalance between the number of features and available samples. Therefore, we utilize a number of topology metrics, widely used for characterising complex networks [5–7] a network of chemicals linked by chemical reactions, and the Internet, a network of routers and computers connected by physical links. While traditionally these systems have been modeled as random graphs, it is increasingly recognized that the topology and evolution of real networks are governed by robust organizing principles. This article reviews the recent advances in the field of complex networks, focusing on the statistical mechanics of network topology and dynamics. After reviewing the empirical data that motivated the recent interest in networks, the authors discuss the main models and analytical tools, covering random graphs, small-world and scale-free networks, the emerging theory of evolving networks, and the interplay between topology and the network's robustness against failures and attacks. A. Quantities of interest in percolation theory 60 B. General results 60 1. The subcritical phase (p\u03fdp c, appropriately generalised for weighted graphs $G := (V, E)$ with |E| vertices and edges:

a. Mean, standard deviation and maximal values of edge weights.
b. Mean, standard deviation and maximal values of vertex degree.
c. Mean, variance and maximal values of shortest path lengths.
d. Alpha-centrality
e. Mean, variance, and maximal values of the page rank.
f. The distance $d(v_i,v_j)$ between the nodes $v_{i,j} \in V$, defined as the sum of the edge weights in the shortest path.
g. The diameter of the graph G as the maximal distance between a pair of vertices.
h. The degree centrality $C_D(G)$ of the graph defined as the normalised graph (G) degree centrality $H(G)$

$$C_D(G) = \frac{H(G)}{H_{max}} \quad (7)$$

which is

$$H(G) = \sum_{i=1}^{|v|} | C_D(v^*) - C_D(v_i) | \quad (8)$$

based on the node degree centrality $C_D(v) = \frac{\deg(v)}{|V|}$, and where v^* is the node with the maximal degree centrality, and $H_{max} = (|V|-1)(|V|-2)$ is the maximal graph degree centrality, obtained for the star topology.

i. Graph or network efficiency [8] $E_c(G)$ defined as

$$E_C(G) = \frac{C_C(G)}{|V|(|V|-1)} \quad (9)$$

based on the graph centrality measure $C_c(G) = \sum_{i \neq j} \frac{1}{d(v_i, v_j)}$

j. Betweenness centrality $C_B(vk)$ of a node as the number of the shortest paths the particular node belongs to:

$$C_B(vk) = \sum_{k \neq i \neq j} \frac{\sigma_{v_i v_j (vk)}}{\sigma_{v_i, v_j}} \quad (10)$$

where $\sigma_{vi,vj}$ is the number of the shortest paths between the nodes v_i and v_j, among which $\sigma_{vi,vj}(vk)$ passing through vk.

k. Google maximal page rank index.

l. Robustness. This is the number of nodes that need to be removed for the network to be unconnected.

These quantities in one way or another should reflect the expected differences between the sample classes. For instance, increasing separation of data sets produces greater edge weights and may result in substantial decrease of the graph diameter. Likewise, nodes with large centrality scores signify their key role in class distinction and give us information on biological importance.

5. Topological indexes are calculated for networks at a range of thresholds (see 3. d.). The threshold at which each topological index provides the best classification of cases and controls (by AUC) in set II are collated and used to generate logistic regression models.

6. The best combination for logistic regression is chosen by comparing all possible combinations. To avoid overfitting the number of indices included in the model is found by forward-backward elimination or limited by a threshold. Categorical variables can be added as extra features for generating the logistic regression.

Supplementary information 2: generation of synthetic data

Data was generated to mimic the behaviour of ovarian cancer biomarkers obtained in the UKCTOCS screening trial [9]. The best biomarker currently used for ovarian cancer detection is CA125, and has been shown to follow a Bayesian Hierarchical Changepoint model [10]. The model is based on the principle that each woman without cancer has her own baseline level of CA125 and all her CA125 values fluctuate around this level. Assume that we have a set of N controls indexed as $i = 1,\ldots,N$.

The number of samples collected for each control is not necessarily the same so we use k_i to denote the number of data points for the i-th patient. The j-th sample for the i-th patient is denoted $Y_{i,j}$ and is taken at $Age_{i,j}$. The model for controls is specified as $Y_{i,j} \sim N(\theta_i, \theta^2)$. with constant mean $E(Y_{i,j} \mid Age_{i,j}) = \theta_i$ and variance σ^2. Previous studies indicate that 85% of cases of ovarian cancer generate a rise in CA125. Therefore the parameter I_i is included in the model for cases, which indicates whether a particular case produces an elevated CA125 ($I_i = 1$) or not ($I_i = 0$). The model for cases with $I_i = 0$ coincides with the model for controls, while in cases with increased CA125, marker levels increase sharply over time after the change-point τ_i at rate γ_i. Thus the mean for cases is modeled by a piecewise linear function:

$$E\left(Y_{i,j|Age_{i,j},I_i=1}\right) = \theta_i + \gamma_i (Age_{i,j} - \tau_i)^+$$

where $(...)^+$ is the positive part of the expression.

Three artificial biomarkers were generated: one followed a hierarchical distribution with the same parameters and prior distributions as for CA125 and two others followed the same distributions with slightly different priors. All parameters are fully described in [10].

Controls were generated from $N(\theta_i, \sigma^2)$, where $\theta_i \sim N(\mu_\theta, \sigma_\theta^2)$, $\mu_\theta \sim N(m_\theta, v_\theta^2)$, $\sigma_\theta^2 \sim IG(a_\theta, b_\theta)$, $\sigma^2 \sim IG(a,b)$, Here m_θ = {2.75,3.05,3.72}, v_θ = {0.1,0.082,0.04}, a_θ = {2.04,2.04,2.04}, b_θ= {0.065,0.065,0.065}, a = {13.95,13.95,13.95}, b = {0.97,0.97,0.98}, for artificial CA125 and two other artificial markers correspondingly. Biomarker behaviour in cases is the same as in controls apart from some extra parameters: $\log(\gamma_i) \sim N(\mu_\gamma, \sigma_\gamma^2)$, $\mu_\gamma \sim N(m_\gamma, v_\gamma^2)$, , and probability of a changepoint π_i. Here, $\mu_\gamma = \{0.4, 0.248, 0.218\}$, $\theta_\gamma^2 = 0.165, 0.172, 0.141$, $a_\gamma = \{2.8, 2.8, 2.8\}$, $b_\gamma = \{1.58, 1.58, 1.58\}$, $\pi_i = \{0.85, 0.75, 0.65\}$ for the three markers respectively.

Based on these distributions, we have generated serial data for 3 artificial biomarkers: one mimics CA125 and two others mimic possible ovarian cancer biomarkers. 27 other "random" analytes were also generated. A further 9 categorical variables were also included: 1 was predictive in 80% of cases, 2 were predictive in 60% of cases with the rest having no disease-association. In total, 300 patients were generated, including 150 cases and 150 controls. Every patient had 9 annual serial measurements (within a 9 year window) for each of the artificial biomarkers.

REFERENCES

1. Venables WN, Ripley BD. Modern Applied Statistics with S. Springer; 2002.
2. Zanin M, Alcazar JM, Carbajosa JV, Paez MG, Papo D, Sousa P, Menasalvas E, Boccaletti S. Parenclitic networks: uncovering new functions in biological data. Sci Rep. Nature Publishing Group. 2014; 4:5112. https://doi.org/10.1038/srep05112.
3. Karsakov A, Bartlett T, Ryblov A, Meyerov I, Ivanchenko M, Zaikin A. Parenclitic Network Analysis of Methylation Data for Cancer Identification. Ruan J, editor. PLoS One. Public Library of Science. 2017; 12:e0169661. https://doi.org/10.1371/journal.pone.0169661.
4. Huberty CJ, Huberty, J. C. Mahalanobis Distance. Wiley StatsRef: Statistics Reference Online. Chichester, UK: John Wiley & Sons, Ltd. 2014. https://doi.org/10.1002/9781118445112.stat06485.
5. ka Albert R, szló Barabá si AL. Statistical mechanics of complex networks. 2002. Available from http://barabasi.com/f/103.pdf.
6. Boccaletti S, Latora V, Moreno Y, Chavez M, Hwang D. Complex networks: Structure and dynamics. Phys Rep. 2006; 424:175–308. https://doi.org/10.1016/j.physrep.2005.10.009.
7. Freeman LC. Centrality in social networks conceptual clarification. Soc Networks. North-Holland. 1978; 1:215–39. https://doi.org/10.1016/0378-8733(78)90021-7.
8. Latora V, Marchiori M. Efficient Behavior of Small-World Networks. Phys Rev Lett. 2001; 87:198701. https://doi.org/10.1103/PhysRevLett.87.198701.
9. Jacobs IJ, Menon U, Ryan A, Gentry-Maharaj A, Burnell M, Kalsi JK, Amso NN, Apostolidou S, Benjamin E, Cruickshank D, Crump DN, Davies SK, Dawnay A, et al. Ovarian cancer screening and mortality in the UK Collaborative Trial of Ovarian Cancer Screening (UKCTOCS): a randomised controlled trial. Lancet. Elsevier. 2015; 387:945–56. https://doi.org/10.1016/S0140-6736(15)01224-6.
10. Skates SJ, Pauler DK, Jacobs IJ. Screening Based on the Risk of Cancer Calculation from Bayesian Hierarchical Changepoint and Mixture Models of Longitudinal Markers. Journal of the American Statistical Association. Taylor & Francis, Ltd.American Statistical Association. 429–39. https://doi.org/10.2307/2670281.

Supplementary Table 1: Demographics and clinical pathology of the ovarian cancer cohort

	Cases	Controls	*P*
No. individuals	30	30	
No. samples	59	59	
Median age at sample draw–yrs (range)	65.2 (50.6–79.7)	64.6 (51.4–78.6)	n.s.
Median time to spin–hrs (range)	22.1 (2.6–46.8)	22.6 (1.7–47.9)	n.s.
Median time to diagnosis–months (range)	14.3 (2.9–97.5)		
Median BMI at recruitment–kg/m^2 (range)	25.0 (19.2–43.2)	24.8 (20.2–40.1)	n.s.
HRT use at recruitment (no.)	6	8	n.s.
OCP use at recruitment (no.)	12	18	n.s.
**Grade and morphology**			
High grade serous	23		
High grade endometrioid	3		
High grade not specified	3		
High grade carcinosarcoma	1		
**FIGO Stage**			
I	7		
II	9		
III	14		

Not significant, n.s..

Supplementary Table 2: Demographics and clinical pathologies of ovarian cancer cases, separated into late (<14.5 months to diagnosis) and early (>34.5 months to diagnosis) groups

	Cases (Late)	Cases (Early)	*P*
No. individuals	30	29	
No. samples	30	29	
Median age at sample draw–yrs (range)	66.9 (55.8–79.7)	62.3 (50.6–55.8)	n.s.
Median time to diagnosis–months (range)	3.8 (2.86–14.3)	58.7 (34.9–97.5)	n.s.
Median BMI at recruitment–kg/m^2 (range)	25.1 (19.2–43.2)	25.0 (19.2–43.2)	n.s.
HRT use at recruitment (no.)	6	6	n.s.
OCP use at recruitment (no.)	12	12	n.s.
**Grade and morphology**			
High grade serous	23	22	
High grade endometrioid	3	3	
High grade not specified	3	3	
High grade carcinosarcoma	1	1	
**FIGO Stage**			
I	7	7	
II	9	9	
III	14	14	

Not significant, n.s..

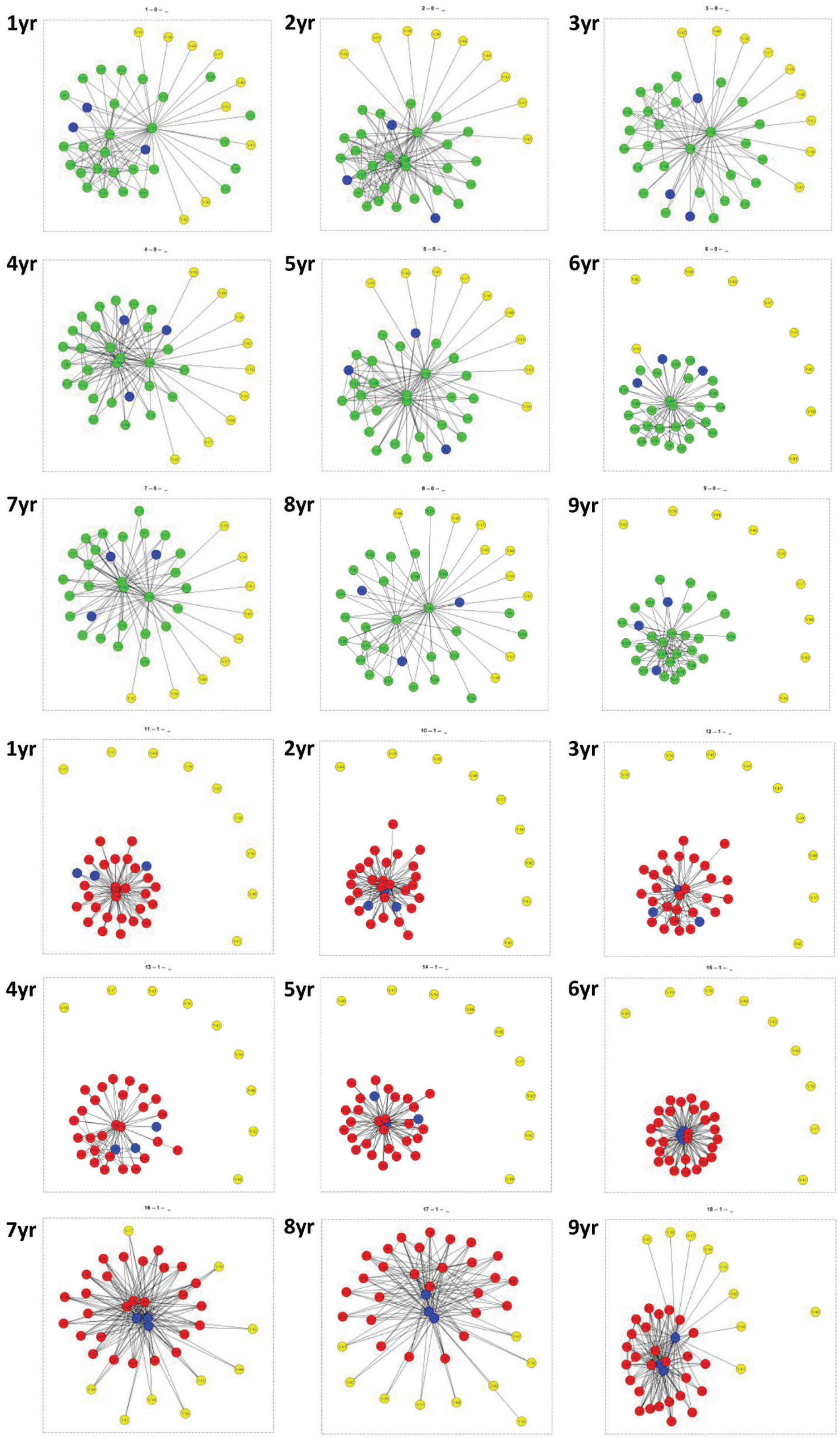

Supplementary Figure 1: Parenclitic networks were generated for "controls" and "cases" from a synthetic data set. From years 1–4, all markers behaved randomly. At year 5, cases developed cancer and markers were modelled for a further 4 years. One marker in the data set mimicked CA125 and 3 others were partially predictive (blue nodes) (for full description, see Supplementary Information). Yellow nodes represent categorical data, green nodes are random markers in controls and red nodes are random markers in cases. Presented is a representative case and control with networks generated from years 1–9. In cases, but not controls, there is an increase in the number of connected nodes after the onset of cancer, furthermore, predictive nodes (blue) become more heavily connected and move towards the centre of the networks.

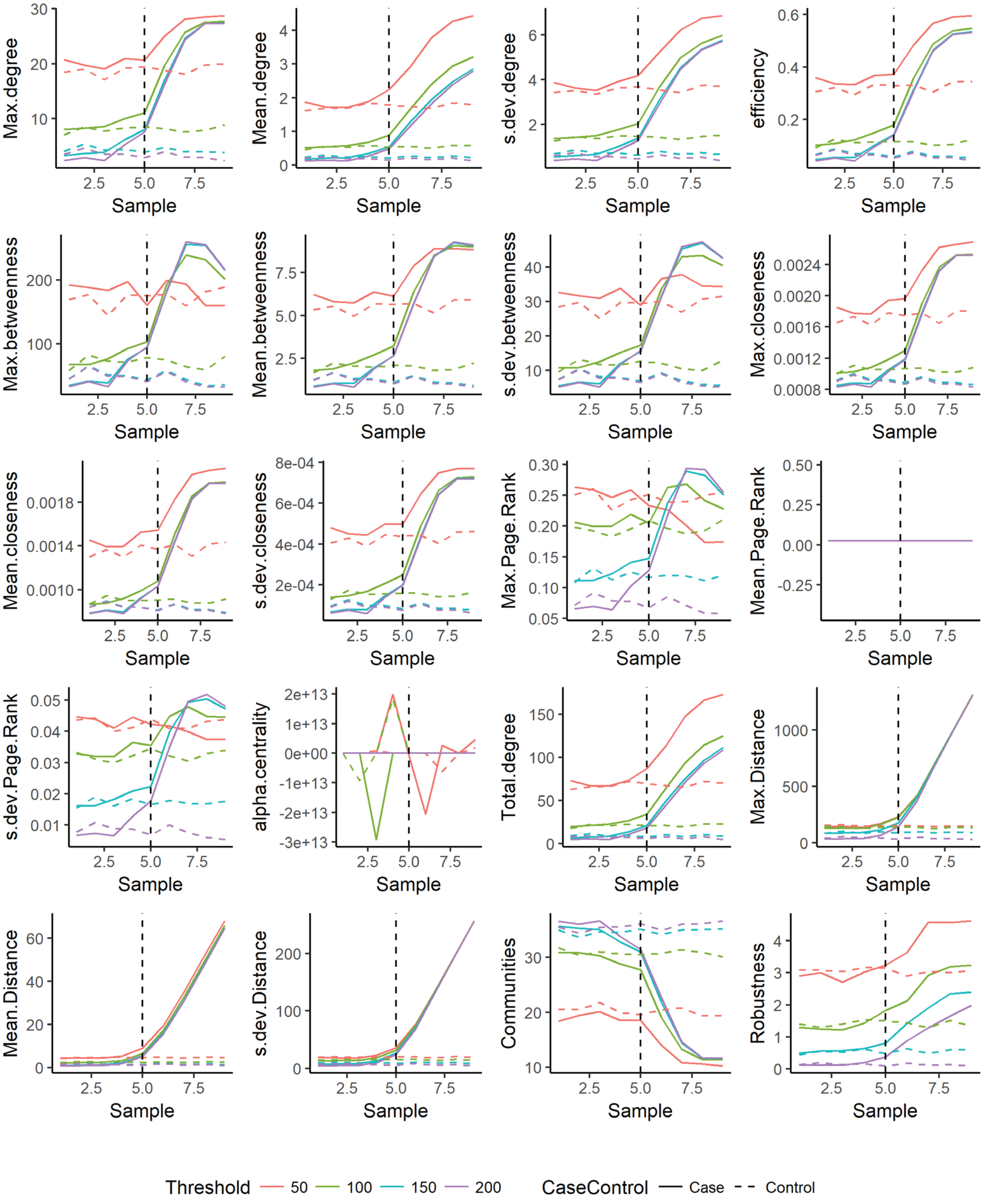

Supplementary Figure 2: Parenclitic networks generated from synthetic data were described by 20 topological indexes at 4 thresholds. Each individual had 9 samples with cancer being initiated in cases at year 5 (vertical dashed line). Presented are the mean topological value for each individual over their 9 samples. Cases are shown as solid lines and controls as dashed lines, colours indicate different thresholds. For further information as to how the thresholds are applied and indices generated, see Supplementary Information. At the initiation of "cancer" in cases, topological features change. For some thresholds, the magnitude of the change is different and thus there is no single threshold that fits all topological features.

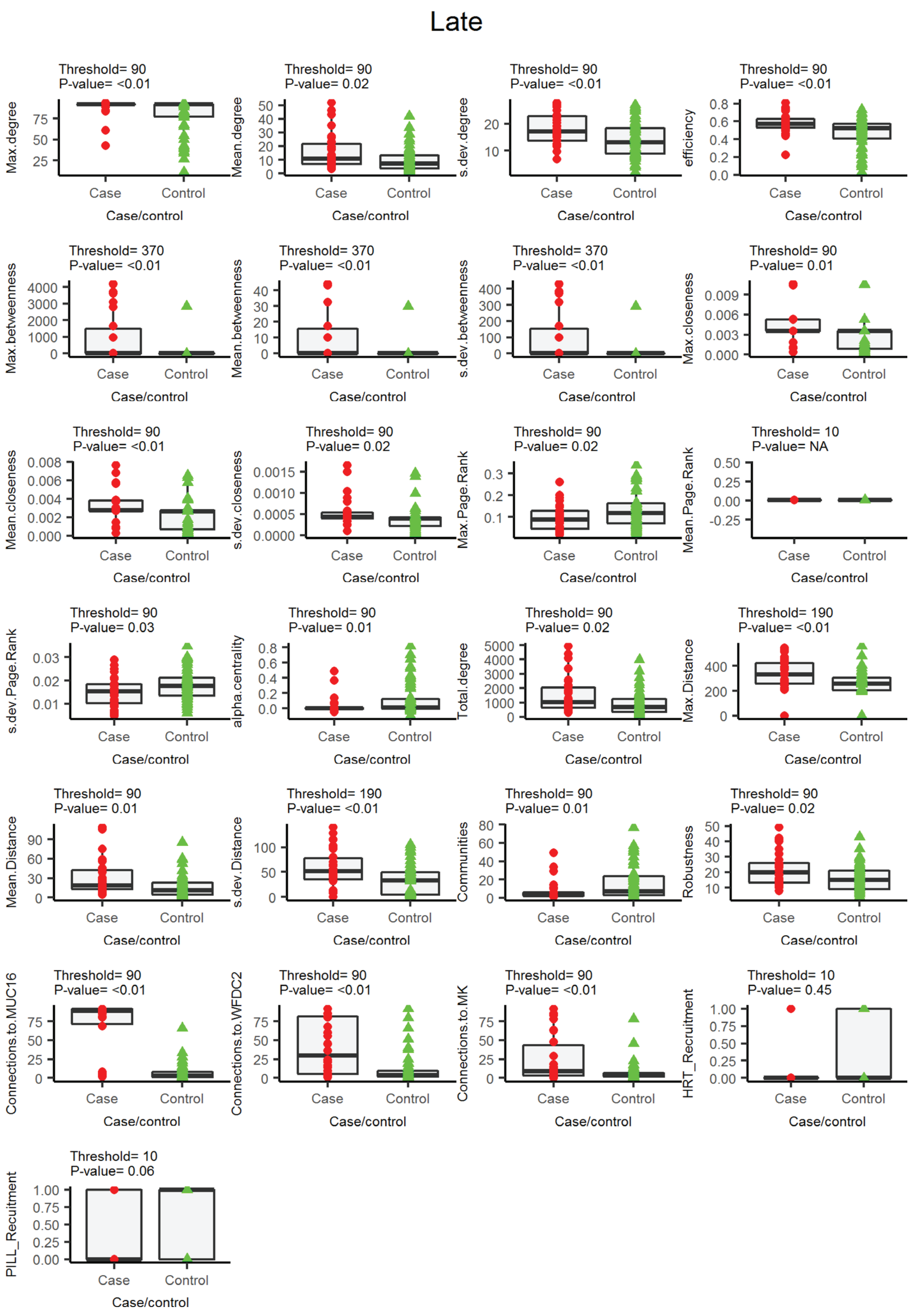

Supplementary Figure 3: In late samples (<14.5 months to diagnosis), parenclitic analysis was performed across a range of thresholds and each network described by a number of topological features. The best threshold for each topology was determined by comparing AUC values. This figure shows the topology values at its optimal threshold for cases and controls from the OC data set. *P*-values were determined by Wilcoxon test.

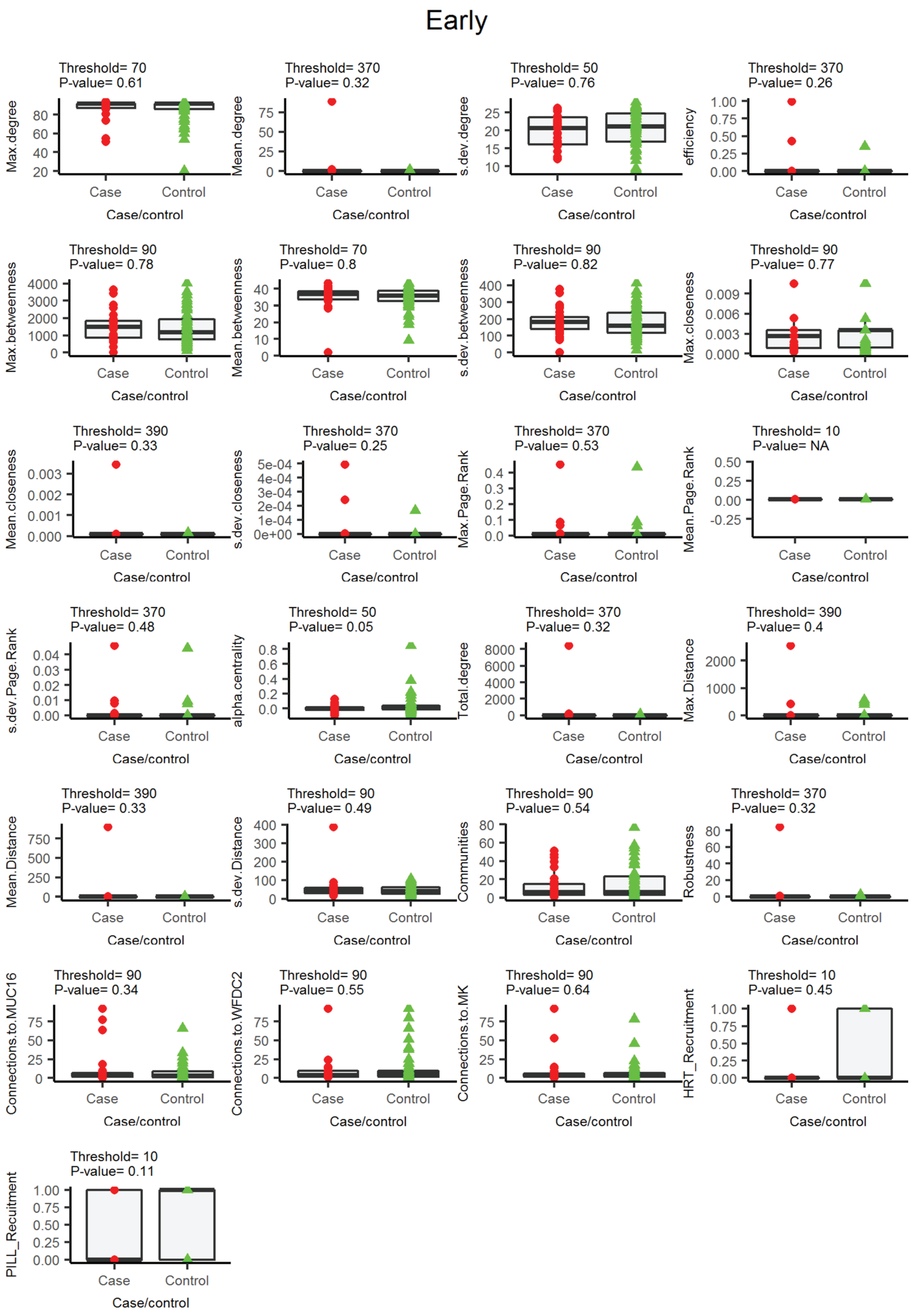

Supplementary Figure 4: In early samples (>36 months to diagnosis), parenclitic analysis was performed across a range of thresholds and each network described by a number of topological features. The best threshold for each topology was determined by comparing AUC values. This figure shows the topology values at its optimal threshold for cases and controls from the OC data set. *P*-values were determined by Wilcoxon test.
